# The Causation and Pathology of Rickets

**Published:** 1892-05-14

**Authors:** 


### The Practitioner's Mirror and Retrospect.

[*The Editor will be glad to receive offers of co-operation and contributions from members of the profession. All letters should be addressed to* The Editor, The Lodge, Porchester Square, London, W.]

### MEDICINE.

### THE CAUSATION AND PATHOLOGY OF RICKETS.

One might think, from the statements made in certain text-books of surgery, that the question of rickets was finally and definitely settled. Absence of lime salts from insufficient or improper food, and consequent inability of the epiphyses to develope normally and join uniformly to the shafts—this, expanded a little, is the usual account given. On consideration, however, there are many problems connected with this important disease which still call for answers. If it is due to a deficiency of phosphate of lime, how is it that calcification of the matrix of the cartilage, and even of the cartilage cells, is so unusually pronounced? If imperfect food is the cause, why should it ever occur amongst well fed and well cared for children? Is it a general or a local disease? Why should the ultimate result be a real increase of the powers of ossification possessed by the ends of the long bones?

Some of these questions may, perhaps, be answered by a review of the facts of the disease.

Rickets is, as everyone knows, very prevalent in the large towns and cities of England and Germany, but is comparatively rare in some countries. For instance, Dr. Jefferis Turner states that only about 5 per cent. of the children attending at the hospital at Brisbane show any signs of it; whereas Dr. Ritter gives 31 per cent. as the proportion of children affected with rickets at the Prague hospitals. Mr. Edmund Owen says that in London the per centage is 30. Now, in spite of Mr. Froude's Utopian account of the large Australian towns, we cannot doubt that the poorer classes suffer from want and imperfect housing as they do elsewhere. Dr. Turner thinks that this superior immunity may be ascribed partly to the increased chance of good food after the second year, and partly to the warmer climate and greater exposure to fresh air and light. It must be granted that the method of building houses in England, especially in crowded cities, is calculated to give the minimum of these two important health factors, viz., air and light, and children can no more thrive without them than plants can. Dr. Muskett (of Sydney) considers that the warmer climate of Australia enables the proteid and carbo-hydrate food to supply actual nourishment to the growing tissues instead of giving its energy to supply body-heat. This seems a very probable theory, but cannot be considered a sufficient explanation, as some very cold climates are exempt from this disease. Apart from the fœtal form of rickets, which is not so common, the symptoms manifest themselves most frequently between the first and second years. This fact has been used as an argument against the supposed hereditary nature of this affection; but there is no reason why a certain inherited condition should not be developed subsequent to birth, and it must be remembered that the epiphyses do not commence their most active period of life until the end of the first or second years, with the exception of the contiguous ends of the femur and tibia. So that we should not expect the disease to manifest itself before this time. That syphilis and tuberculosis in the parents is calculated to produce rickety children is probable, not because there is any connection between the diseases, but because any influence which weakens the offspring may, indirectly, tend to cause imperfect bone formation.

As to the actual changes which go on in the ends of the long bones, it would appear that there is rather a perversion of activity than a diminution. There is an excessive multiplication of the cartilage cells, which soon wither and assume irregular forms, many of them resembling in shape connective tissue cells. There is a greatly increased amount of calcification, but the process seems to make little advance beyond this point. The activity of the cells seems to exhaust the part, and the subsequent deposition of lime salts is probably a sign of degeneration. Pepper ("Surgical Pathology") remarks that "anything that tends to diminish the vitality of the tissues predisposes to calcification." An examination of the end of a rickety bone shows the above; and irregular patches of true spongy bone may be seen developing, not from one point, as is the normal condition, but from numerous centres. These are generally surrounded by a plentiful supply of blood vessels and small cells of irregular forms. These foci ultimately enlarge and join together so that in the long run there is really more bone formed than in the healthy process. This is interesting in connection with a suggestion thrown out by an eminent pathologist, that bone formation is really a form of tissue degeneration. On the whole facts point to the conclusion that the disease is, as stated above, a perversion of metabolism, rather than an arrest of growth; that any bad hygienic condition may increase it, although its starting point is determined by obscure causes, of which we are as yet ignorant. The "bad conditions" referred to may be from food, climate, want of light and fresh air, &c., and transmitted frailty of the organism is no doubt an important factor.

